# Experimental and Numerical Investigation of Bogie Hunting Instability for Railway Vehicles Based on Multiple Sensors

**DOI:** 10.3390/s24124027

**Published:** 2024-06-20

**Authors:** Biao Zheng, Lai Wei, Jing Zeng, Dafu Zhang

**Affiliations:** State Key Laboratory of Rail Transit Vehicles System, Southwest Jiaotong University, Chengdu 610031, China; zhengb1991@163.com (B.Z.); zeng@swjtu.edu.cn (J.Z.); feng20065193@163.com (D.Z.)

**Keywords:** bogie hunting, high conicity, multiple sensors, filed test, brake caliper, geometric interference

## Abstract

Bogie hunting instability is one of the common faults in railway vehicles. It not only affects ride comfort but also threatens operational safety. Due to the lower operating speed of metro vehicles, their bogie hunting stability is often overlooked. However, as wheel tread wear increases, metro vehicles with high conicity wheel–rail contact can also experience bogie hunting instability. In order to enhance the operational safety of metro vehicles, this paper conducts field tests and simulation calculations to study the bogie hunting instability behavior of metro vehicles and proposes corresponding solutions from the perspective of wheel–rail contact relationships. Acceleration and displacement sensors are installed on metro vehicles to collect data, which are processed in real time in 2 s intervals. The lateral acceleration of the frame is analyzed to determine if bogie hunting instability has occurred. Based on calculated safety indicators, it is determined whether deceleration is necessary to ensure the safety of vehicle operation. For metro vehicles in the later stages of wheel wear (after 300,000 km), the stability of their bogies should be monitored in real time. To improve the stability of metro vehicle bogies while ensuring the longevity of wheelsets, metro vehicle wheel treads should be reprofiled regularly, with a recommended reprofiling interval of 350,000 km.

## 1. Introduction

Hunting stability is a crucial aspect of railway vehicle operational safety. Due to the conicity of wheel treads, according to Klingel’s theory, wheels exhibit a combined lateral and yaw movement along the track’s centerline with a certain wavelength, known as hunting motion [1]. Under normal conditions, when a vehicle operates below critical speed, hunting motions induced by disturbances typically stabilize quickly to an equilibrium position. However, if the vehicle exceeds the critical speed, disturbances can lead to periodic oscillations with increasing amplitude, resulting in instability, which is referred to as hunting instability [2,3,4]. In real-world operations, hunting stability is influenced by various factors such as wheel–rail wear, track geometry, curve radius, track gauge, and suspension system, which means instability can occur even at speeds below the critical speed. The hunting motion of the vehicle system can be divided into carbody hunting and bogie hunting [5]. Carbody hunting usually occurs in a low wheel–rail conicity manifested as low-frequency swaying of the body, known as carbody sway. Carbody hunting not only affects ride comfort but can also threaten vehicle operational safety in certain circumstances [6,7,8,9]. On the other hand, when the equivalent conicity of the wheel–rail contact is too high, the vehicle may experience bogie hunting, characterized by high-frequency vibrations of the bogie frame. Bogie hunting instability can lead to fatigue failure of the frame [10], a sharp deterioration in vehicle operational safety indicators, and pose a threat to vehicle operational safety [11,12].

Researchers have conducted extensive studies on the bogie hunting instability of railway vehicles. Bustos [13] established a nonlinear model of the Spanish high-speed train and investigated the hunting stability of the vehicle using the root locus method. Additionally, a sensitivity analysis of the suspension parameters was performed. Skerman [14] utilized Gensys software to construct a three-piece bogie model for freight trains and conducted simulation calculations using both acceleration and deceleration methods to determine the critical speed of the vehicle under the U.S. Class 6 spectrum track excitation. Uyulan [15] established a 12-degree-of-freedom bogie model and an 8-degree-of-freedom dual wheelset model and utilized Lyapunov’s indirect method to investigate the bifurcation characteristics on curved tracks. To enhance the hunting stability of railway vehicles, methods such as electrically adjustable suspension, active and semi-active control, and adaptive suspension parameters have been proven to be feasible [16,17,18,19].

In recent years, numerous scholars have applied sensors to railway vehicle systems to monitor and identify faults during vehicle operation [20,21,22,23,24,25]. Given the numerous hazards associated with bogie hunting instability, it has become increasingly important to monitor bogie hunting instability by deploying sensors on vehicles. In China, by installing acceleration sensors at the ends of the high-speed EMU bogie frame, the data recorded by the acceleration sensors is processed in real time. After filtering the lateral acceleration, if it exceeds 8 m/s^2^ six consecutive times, the onboard alarm system is triggered, and the vehicle will run at a reduced speed [26]. According to the European TSI standard [27], if the lateral acceleration signal at the bogie frame ends, after being filtered in the 3–9 Hz range, reaches or exceeds the threshold of 0.8 g ten consecutive times, it is determined to be bogie lateral instability.

Based on the lateral acceleration of the bogie frame, some scholars have studied various methods for monitoring bogie hunting instability. By placing acceleration sensors on the bogie frame and the carbody, Li [28] investigated the monitoring and suppression measures of bogie instability in high-speed EMUs. The research results showed that reprofiling wheels, replacing anti-hunting dampers, and grinding rails could suppress bogie instability. Kulkarni [29] studied the correlation between the lateral and longitudinal acceleration of the axle box and the vehicle’s hunting motion, proposing an index that combines the phase and vibration amplitude of lateral and longitudinal axle box acceleration to assess whether the vehicle experiences hunting instability. Sun [30] collected the lateral acceleration signals at the ends of the bogie frame and used a singular value decomposition method based on the Hankel matrix to predict wheelset lateral displacement and yaw angle. The effectiveness of this method was validated via comparisons with simulation results and test bench experiments, but it has not been validated in field tests. Ning [31] proposed a method based on multi-scale permutation entropy and local tangent space alignment for the small amplitude hunting motion of high-speed trains at speeds of 320–350 km/h. Currently, monitoring and identifying bogie instability mainly focuses on high-speed trains, while metro vehicles, which operate at lower speeds, often lack attention. Furthermore, these monitoring methods are primarily based on the acceleration of the bogie frame or axle box. However, from the perspective of operational safety, the axle force more directly reflects the impact of bogie hunting on the safety of vehicle operation, and the aforementioned methods evidently cannot provide an assessment of axle force. For the wheelset motion posture under bogie instability, the correlation between wheelset lateral displacement and yaw angle with the bogie frame acceleration can only be established via simulation and lacks broad applicability.

The brake caliper is suspended from the bogie frame, converting brake air pressure from the brake cylinder into a normal force applied to the brake disc by the brake pads, thereby achieving vehicle braking. The proper functioning of the brake caliper ensures safe braking of the vehicle within an appropriate distance. However, due to bogie hunting instability, there can be geometric interference between the brake caliper and the wheelset, potentially damaging the brake caliper and compromising the operational safety of metro vehicles. Past studies have mainly focused on assessing the brake caliper’s reliability under braking conditions. However, research on the vibration and dynamic behavior of the brake caliper during non-braking conditions has been limited. There is a notable research gap concerning the vibration characteristics of brake calipers under non-braking conditions, especially concerning the geometric interference between the brake caliper and the wheelset during lateral vibrations.

The aim of this study is to monitor bogie hunting instability in metro vehicles and the geometric interference between the brake caliper and the wheelset using multiple sensors. It entails analyzing the root causes and remedies for bogie hunting instability in metro vehicles via field tests and numerical simulations. The structure of the subsequent sections is as follows: Section 2 introduces the selection and positioning of acceleration and displacement sensors, along with the methodology and procedures of field testing. Section 3 presents the findings of the field tests and examines the acceleration and displacement signals collected. Additionally, this section evaluates the impact of bogie hunting instability on the safety and ride comfort of metro vehicles. Section 4 commences by establishing a metro vehicle dynamics model, which includes the brake caliper. Subsequently, the accuracy of the dynamics model is validated by comparing it with field test data. Finally, a simulation analysis is carried out to address the geometric interference between the brake caliper and the wheelset caused by bogie hunting instability, focusing on the stiffness of the brake caliper suspension and the wheel–rail contact relationship.

## 2. Sensors and Methods

In response to the bogie hunting instability caused by high conicity wheel–rail contact of metro vehicles, this section employs a method of conducting field tests using multi-sensors to assess vehicle vibration characteristics. Firstly, suitable sensors are selected based on the measured objects, followed by an introduction to the testing methodology and the experimental process.

### 2.1. Selection of Sensors

To test the vibration transmission characteristics under bogie hunting instability in metro vehicles, acceleration sensors are used to measure the vibration acceleration in three directions: axle box, bogie frame, and carbody floor. For accuracy in measurement and equipment safety, the measurement range of the acceleration sensor should match the measured acceleration amplitude. According to the relevant literature in the introduction section, acceleration sensors with a range of 5 g (1 g = 9.81 m/s^2^) are chosen for the carbody floor, while 18 g and 100 g are selected for the bogie frame and axle box, respectively. It should be noted that sensors with larger measurement ranges have lower accuracy, so using sensors with a large measurement range to test small acceleration values can lead to a loss of precision in the test results.

Due to bogie hunting instability of metro vehicles, the brake calipers may interfere with the wheelsets during lateral vibrations. The relative displacement between the frame and wheelset, as well as between the brake caliper and wheelset, needs to be tested using displacement sensors. For the measurement of relative displacement, laser displacement sensors with higher accuracy are employed. Unlike acceleration sensors, the selection of laser displacement sensors not only considers the measurement range but also the relative distance of the measured objects. The relative displacement between the frame and wheelset is obtained by measuring displacement in three directions (longitudinal, lateral, and vertical) of the primary suspension, for which a 30–130 mm range laser displacement sensor is chosen. Due to the relatively large distance between the bottom of the brake caliper and the wheelset, a larger range laser displacement sensor of 50–300 mm is selected for measuring the relative displacement between the brake caliper and the wheelset.

The selected sensor parameters are shown in Table 1.

### 2.2. Layout of Sensors

**Figure 1:**
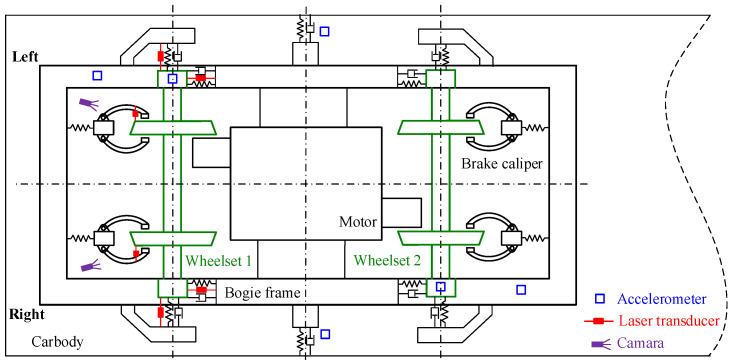
Sensor layout points for field test.

As shown in Figure 1, the schematic diagram illustrates the installation positions of various sensors. Moving to the left indicates the forward direction, with the vehicle’s left and right sides defined accordingly. Blue squares represent the accelerometer sensors. Accelerometer sensors are positioned on the left and right axle boxes of wheelset 1 and wheelset 2, respectively. Two accelerometer measurement points are diagonally located at the left front and right rear corners of the frame. Additionally, accelerometer sensors are installed on the carbody floor to measure carbody acceleration. All accelerometer sensors are triaxial, measuring acceleration in the longitudinal, lateral, and vertical directions, respectively. Red squares denote the laser displacement sensors. Three laser displacement sensors are installed on both sides of wheelset 1 to measure longitudinal, lateral, and vertical displacements of primary suspension. Laser displacement sensors are also placed on both sides of wheelset 1 to measure the lateral relative displacement between the brake caliper and the wheelset. To monitor the relative motion between the brake caliper and wheelset, two cameras are mounted on the frame to observe the motion status of the brake caliper on both sides of wheelset 1. The cameras are represented by purple squares. Additionally, the bottom right corner of Figure 1 shows the three-dimensional model of the brake caliper.

**Figure 2:**
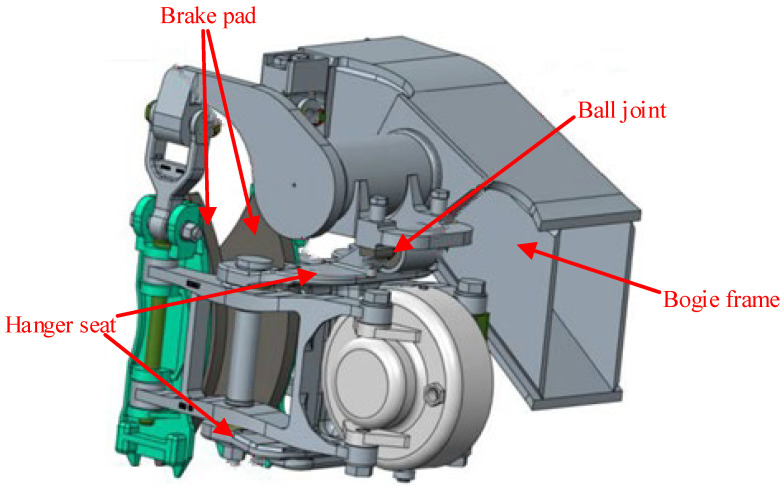
Brake caliper suspended on the frame.

Figure 2 shows a schematic of the brake caliper suspended from the frame. The brake caliper is suspended from the frame via a hanger, with a ball joint connection between the brake caliper hanger and the frame. The brake pads are suspended from the frame through hangers, allowing free movement between the hangers and the frame. The geometric interference between the brake caliper and the wheel occurs at the bottom of the hanger seat.

**Figure 3:**
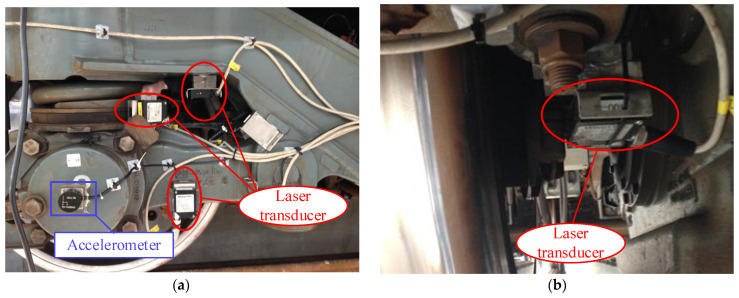

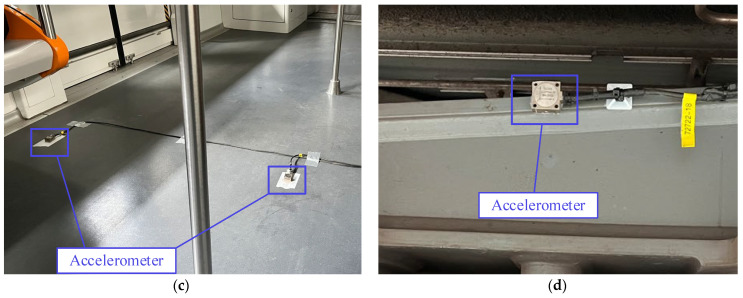
Mounting sensors on the tested vehicle; (**a**) sensors on left of wheelset 1; (**b**) sensor on brake caliper; (**c**) sensors on carbody floor; (**d**) sensor on bogie frame.

Figure 3 shows the installation of sensors on the tested metro vehicle. Figure 3a illustrates the left side of wheelset 1, with three red circles representing the longitudinal, lateral, and vertical laser displacement sensors of the primary suspension. The blue rectangle indicates the accelerometer installed on the axle box. Figure 3b displays the laser displacement sensor mounted on the brake caliper to measure the lateral relative displacement between the brake caliper and the wheelset. Figure 3c illustrates the accelerometer mounted on the vehicle body floor. Figure 3d depicts the accelerometers mounted on the bogie frame.

### 2.3. Field Test

**Figure 4:**
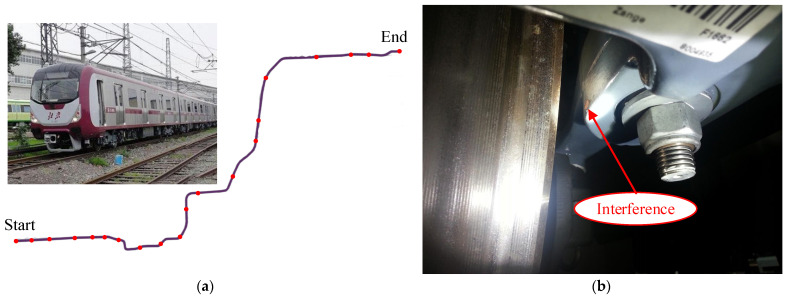

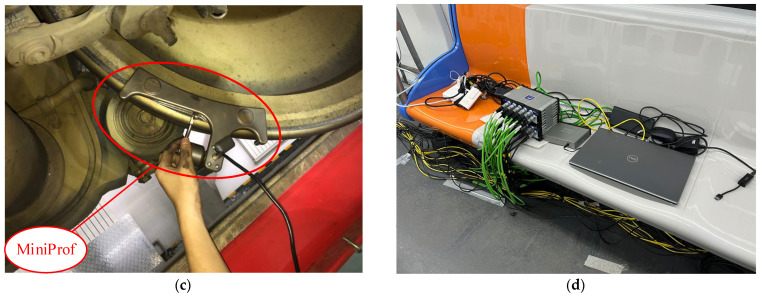
Field test of bogie hunting; (**a**) tested metro vehicle and line; (**b**) interference between brake caliper and wheelset; (**c**) tread profile detection; (**d**) data acquisition equipment.

The tested metro vehicle is equipped with a swing arm and steel springs for primary suspension, along with primary vertical dampers. The secondary suspension comprises air springs and secondary lateral dampers without yaw dampers. Figure 4a depicts the tested metro vehicle and the line. During the test, the tested vehicle departed from the starting point and operated at a maximum speed of 100 km/h on the metro line, which has a total length of 41.4 km. The red dots represent the stations, with a total of 20 stations. As in actual operation, the metro vehicle made a brief stop at each station before accelerating again. From the metro line, it can be seen that there are some small-radius curves in the middle section of the line. When passing through these small-radius curves, the maximum speed of the subway vehicle is limited to 80 km/h. Figure 4b illustrates the geometric interference between the brake caliper and the wheelset caused by bogie hunting instability.

The wheel–rail contact relationship has a significant impact on bogie hunting stability, making an investigation into the wheel–rail relationship essential in the experiment. The wheel tread and rail profile are measured using the MiniProf device. Figure 4c illustrates the measurement of wheel tread data using the MiniProf equipment.

The data collected in this experiment was recorded using the HBM eDAQ device (HBM Sensorik GmbH in Darmstadt, Germany), which features state-of-the-art signal conditioning capabilities, extensive data processing, intelligent data storage, and sophisticated computing capabilities. Figure 4d shows the data acquisition equipment. When connected to a laptop via wired or wireless means, the HBM eDAQ can monitor the vibration characteristics of the vehicle in real time. The data measured by the sensors is saved on the computer in 2 s intervals via the data acquisition system. Each set of data is then processed and reconnected. The data processing method will be introduced in Section 3. Additionally, when connected to the internet, remote monitoring and control can be achieved through a web server. The processed data will be transmitted to the metro company control center through the network, allowing real-time display of the vehicle’s operating status. To ensure the reliability of the experimental data, the sampling frequency for acceleration and displacement signals was set to 2000 Hz during the experiment.

## 3. Results and Analysis

This section analyzes the data collected from the field test. By processing the acceleration signals in the time and frequency domains for the carbody, frame, and axle box, the vibration transmission and frame vibration characteristics under bogie hunting instability are analyzed. Based on the displacement data signals, the relative motion between the brake caliper and wheelset caused by bogie hunting instability is examined.

### 3.1. Wheel–Rail Contact

**Figure 5:**
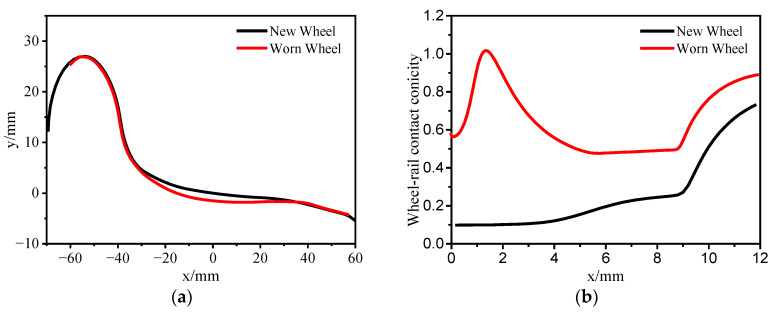
Investigation of wheel–rail contact nonlinearity; (**a**) comparison of new and worn wheel profile; (**b**) contact conicity.

Since the last wheel reprofiling, the vehicle has operated for 380,000 km. The test track uses a CN60 profile with a rail weight of 60 kg/m. The track is ballastless and uses ordinary fasteners instead of vibration-damping fasteners. The track uses the standard gauge of 1435 mm with a rail cant of 1/40. In the wheel–rail contact relationship, the nominal rolling circle distance is set to 1493 mm. Figure 5a shows the comparison between the worn wheel profile and the new wheel profile measured using MiniProf, as reported in Section 2.3. It can be observed from the graph that wear is mainly concentrated in the middle of the wheel profile, forming concave wear, while the wear on the wheel flange is less pronounced. Wheel wear affects the nonlinear relationship between the wheel and rail contact, thereby influencing the lateral stability of the vehicle. Equivalent conicity is an important parameter describing the wheel–rail contact relationship, referring to the rate of change of geometric clearance or distance between the wheel and rail in the lateral direction. Equivalent conicity has significant effects on the safety and ride comfort of railway vehicle operation. Following the calculation method provided by UIC 519 [32], the equivalent conicities of the new and worn wheel profiles were calculated, as shown in Figure 5b. It can be seen from the graph that the equivalent conicity of the new wheel profile is around 0.1 at a lateral shift of 3 mm. However, after wheel wear, the equivalent conicity at a 3 mm lateral shift exceeds 0.6. This high equivalent conicity of the wheel–rail contact relationship is the fundamental cause of bogie hunting instability in metro vehicles.

### 3.2. Vibration Analysis

**Figure 6:**
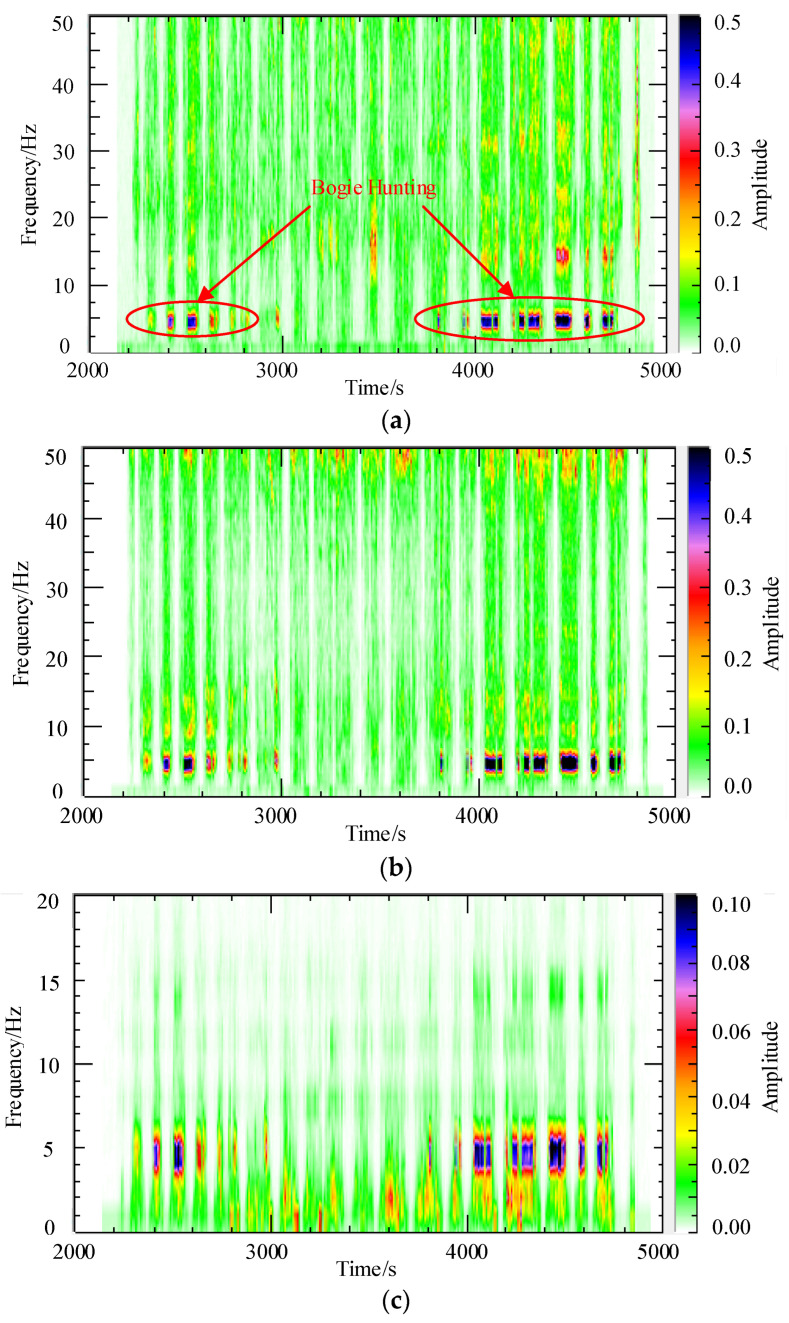
STEFT spectrum of lateral accelerations; (**a**) axle box; (**b**) bogie frame; (**c**) carbody.

Short-time Fourier transform (STFT) is a commonly used method for signal spectrum analysis. It decomposes a signal into frequency components over different time intervals to better understand the signal’s variations in both time and frequency domains. STFT is typically achieved by dividing the signal into multiple short segments and performing Fourier transforms on each segment. By applying STFT to the lateral acceleration signal obtained from the field test in Section 2.3, the resulting spectrograms are shown in Figure 6. Figure 6a–c represent the spectrograms of the lateral acceleration for the axle box, frame, and carbody, respectively. The field test described in Section 2.3 lasted for one and a half hours, during which the data acquisition instruments recorded nearly 5000 s of data. The first 2000 s capture the vehicle moving from the parking area to the metro line and are therefore not considered in the analysis. The acceleration data analyzed in the figures corresponds to the period between 2000 s and 5000 s. From the analysis, it is evident that bogie hunting instability occurred between 2400 s to 2700 s and 3800 s to 4800 s. During these periods, the vehicle system exhibited significant harmonic vibrations, with the vibration main frequency concentrated around 5 Hz. As described in Section 2.3, there are small-radius curves in the middle section of the metro line, and the maximum speed of the vehicle is limited to 80 km/h when passing through these curves. This explains why bogie hunting instability did not occur in the metro vehicle between 3000 and 38,000 s during the field test.

**Figure 7:**
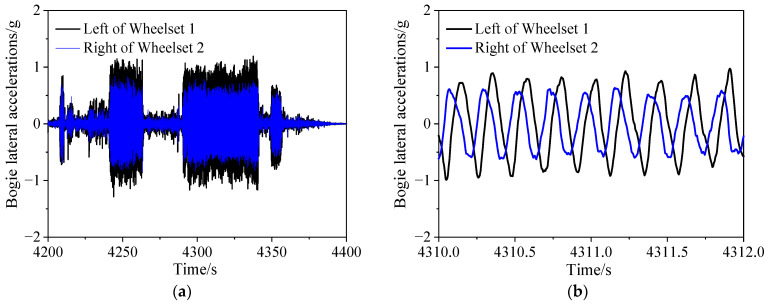
Dynamic evaluation results of tested vehicle; (**a**) wheel axle forces; (**b**) ride index.

Additionally, Figure 7 shows the time-domain plot of the lateral acceleration for the bogie frame. To clearly analyze the harmonic vibrations occurring in the bogie, Figure 7a presents the tested data for the interval between 4200 and 4400 s, while Figure 7b focuses on the data from the interval between 4310 and 4312 s. To avoid phase delay caused by filtering, a fourth-order Butterworth band-pass filter with a cutoff frequency of 0.5 Hz to 10 Hz, according to UIC 518 [33], is applied to the lateral acceleration signal of the frame. It can be seen from the time-domain plot of the frame’s lateral acceleration that the maximum value exceeds 1 g. According to GB5599 [26], bogie lateral instability is determined when the lateral acceleration of the bogie frame, after passing through a 0.5–10 Hz band-pass filter, reaches or exceeds a peak value of 8 m/s^2^ continuously for more than six instances. Figure 7b is a locally magnified view of the acceleration signal, showing the presence of harmonics, with nine waveforms within 2 s, indicating a frame lateral vibration frequency of 4.5 Hz. There is a phase difference between the lateral accelerations at the ends of the frame on the left side of wheelset 1 and the right side of wheelset 2, with a greater magnitude of lateral acceleration at the left end of the frame on wheelset 1. Based on the analysis of the acceleration signal, the motion of the frame is primarily characterized by lateral and yaw movements.

**Figure 8:**
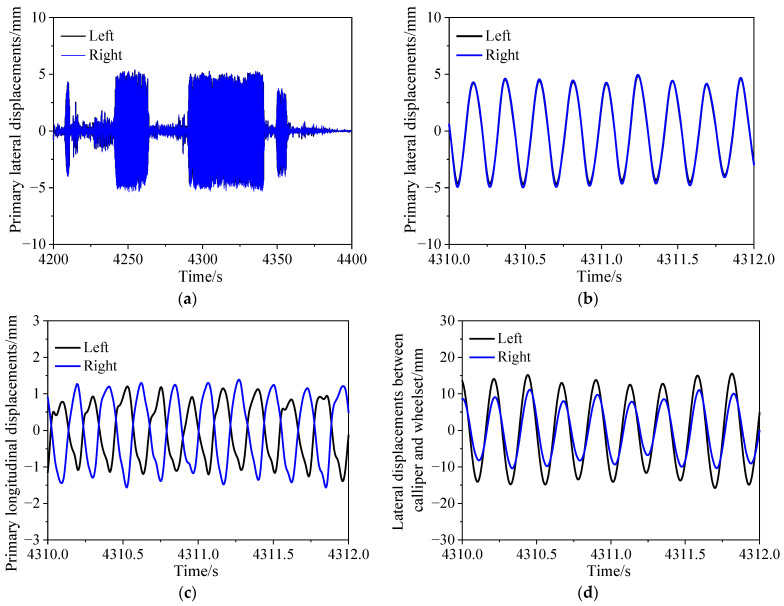
Measured displacements of metro vehicle; (**a**,**b**) primary lateral displacements; (**c**) primary longitudinal displacements; (**d**) lateral displacements between caliper and wheelset.

Figure 8 depicts the time-domain plots of displacement signals obtained during the field tests. Consistent with the method used for acceleration signal processing, a fourth-order Butterworth bandpass filter with a cutoff frequency of 0.5–10 Hz was applied. Figure 8a,b show the time domain of lateral displacement of the primary suspension. It can be observed that the lateral displacement exhibits harmonic oscillations with an amplitude of up to 5 mm. The amplitudes of lateral displacement on the left and right sides of the wheelset are nearly identical, with consistent phases. Figure 8c illustrates the longitudinal displacement of the primary suspension. The waveforms of longitudinal displacement on the left and right sides of the wheelset are out of phase, with amplitudes close to 1.5 mm. Analysis of lateral and longitudinal displacement signals indicates that the predominant motion of the wheelset relative to the primary suspension is lateral and yaw. Considering the large lateral span of the longitudinal measurement points on the left and right sides, the yaw motion of the wheelset is relatively minor. Figure 8d displays the time domain of the relative lateral displacement between the left and right brake calipers and the wheelset. It can be observed that the relative lateral displacement between the left brake caliper and the wheelset is greater than that on the right side, with an amplitude exceeding 15 mm. Since the brake calipers are suspended from the bogie frame, if the brake calipers are considered to be part of the primary suspension, the relative lateral displacement between the brake calipers and the wheelset should be close to the lateral displacement of the primary suspension. The discrepancy between the two is due to the relative motion between the brake calipers and the bogie frame. The video recorded by the cameras during the experiment also confirms this observation. The cameras are fixed on the frame, and the video shows significant lateral swinging motion of the brake caliper.

**Figure 9:**
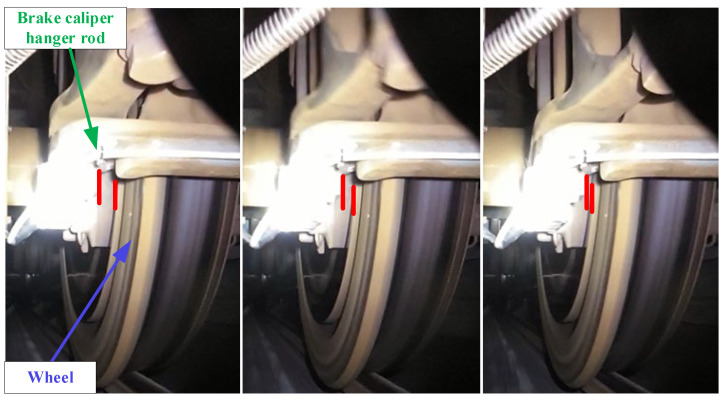
Relative motion between brake caliper and wheel (Red lines indicate the distance between brake caliper and wheel).

Figure 9 illustrates a screenshot from the camera monitoring video. The green arrow in the figure points to the brake caliper hanger, while the blue arrow points to the wheel. The three images on the left, middle, and right are captured sequentially over time. From left to right, the images illustrate the process of the brake caliper hanger swinging from the far left to the far right due to bogie hunting. Two short red lines in the images represent the lateral clearance between the bottom of the brake caliper hanger and the wheel. As shown, the distance between the two red lines decreases, indicating that the lateral clearance between the bottom of the brake caliper hanger and the wheel gradually reduces until geometric interference occurs.

In summary, the high conicity wheel–rail contact resulting from wheel wear leads to bogie hunting instability in metro vehicles. By deploying multi-sensors on metro vehicles and utilizing data acquisition equipment, the issue of bogie hunting instability can be monitored. Bogie hunting instability in metro vehicles manifests as lateral and yaw motion around 5 Hz, which is transmitted to the carbody through the secondary suspension. Additionally, lateral vibrations of the bogie frame cause the brake caliper to swing laterally around the suspension point. This lateral swing of the brake caliper results in significant lateral relative displacement between the bottom of the brake caliper hanger seat and the wheel, with an amplitude of up to 15 mm. Although the nominal clearance between the brake caliper and the wheelset is 17.5 mm, due to assembly errors, the measured clearance range on-site is between 12 mm and 23 mm. This indicates that when the lateral relative displacement between the brake caliper and the wheelset exceeds 12 mm, geometric interference occurs between the brake caliper and wheel.

### 3.3. Dynamic Evaluation

The bogie hunting instability in metro vehicles not only leads to geometric interference between the brake caliper and wheelset but also affects the safety and ride comfort of the vehicles. Currently, the methods for measuring wheel–rail forces are mainly divided into two categories. The first category is direct measurement methods, which involve placing multiple sets of strain gauges on the wheel and axle positions and connecting them in a bridge circuit to achieve dynamic wheel–rail force measurement. This method requires modifications to the wheelsets of the vehicle, which can be costly and time-consuming. The other method is the indirect measurement, which utilizes the lateral and vertical force equations of the wheelset, measuring physical quantities such as primary suspension displacement and axle box acceleration to identify wheel–rail forces. In this study, the wheelset lateral forces are obtained using the indirect measurement method [34]. According to GB5599 [26], the limit value of wheelset lateral force is given by Formula (1). Ride index indicators are divided into vertical and lateral ride index indicators, which are calculated using the vertical and lateral accelerations of the carbody collected during the field test. According to GB5599, the calculation method for ride comfort is given by Formula (2).

(1) $$H\leq 15+P_{0}/3$$

where *H* is the wheelset lateral force, *P*_0_ is the axle load, and the units are kN.

(2) W=3.57A3fFf10

where *W* is the ride comfort index, *A* is the carbody vibration acceleration, *f* is the vibration frequency, and *F*(*f*) is the frequency correction factor, as shown in Table 2.

**Figure 10:**
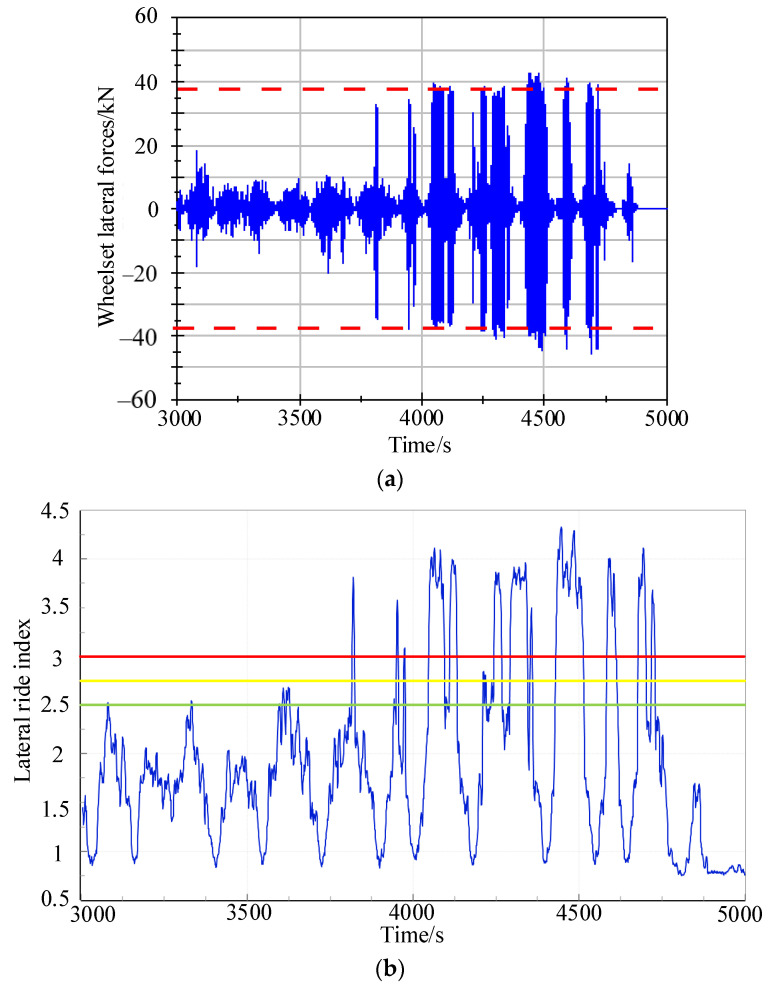

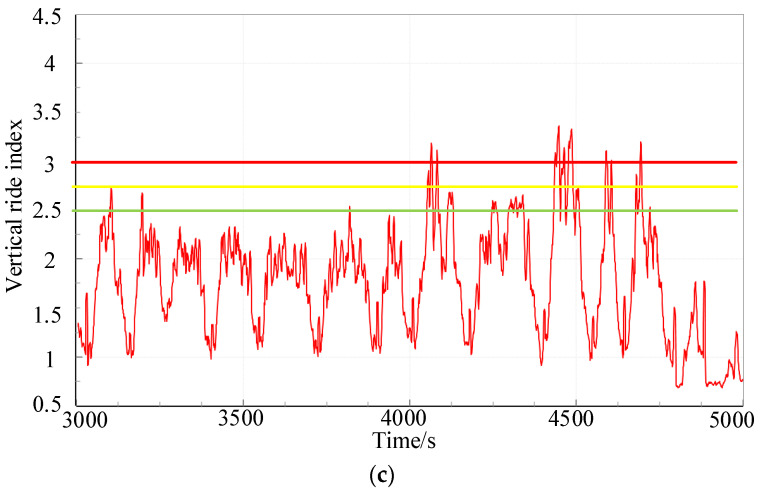
Dynamic evaluation results of tested vehicle; (**a**) wheel axle force; (**b**) lateral ride index; (**c**) vertical ride index.

According to the analysis of the lateral acceleration of the bogie frame in Section 3.2, the tested vehicle experienced bogie hunting instability during the 3800–4800 s interval. Therefore, tested data from the 3000–5000 s interval was selected to calculate the lateral wheelset lateral forces using the indirect measurement method. The wheelset lateral force calculated using the indirect measurement method is shown in Figure 10a. From the figure, it can be observed that within the bogie hunting region, the wheelset lateral force exceeds the safety limit set in GB5599. Figure 10b,c display the lateral and vertical ride index. According to the evaluation criteria for ride index indicators in GB5599, a ride index below 2.5 is considered excellent, between 2.5 and 2.75 is good, between 2.75 and 3.0 is acceptable, and above 3.0 is considered unacceptable. From the ride index graphs, it can be seen that within the bogie hunting instability zone, both lateral and vertical ride indices exceed 3.0. In some sections, the lateral ride comfort indicator even exceeds 4.0.

The analysis above reveals that bogie hunting instability induced by high conicity wheel–rail contact affects the safety of metro vehicle operation. The lateral and yaw motion of the bogie significantly impacts the lateral ride index more than the vertical ride index. When bogie hunting instability occurs, the lateral ride comfort indicator of the vehicle exceeds 4.0.

In summary, using the testing methods described in Section 2, the processed lateral wheel–axle forces and comfort indices can be transmitted in real time to the metro vehicle control center, allowing for real-time monitoring of the vehicle’s operating status. When the monitored lateral wheel–axle forces and comfort indices exceed the limit values, the vehicle’s speed will be reduced to ensure safety. The following sections of this paper will analyze the interference between the brake caliper and the wheel, as well as solutions for metro vehicle hunting instability, through numerical simulation.

## 4. Modeling and Simulation

In this section, the detection of bogie hunting instability caused by high conicity wheel–rail contact is simulated by establishing a dynamic model of metro vehicles, including brake calipers. The simulation follows the sensor layout and testing methods described in Section 2. Based on the vibration characteristics analysis from Section 3.2, solutions are simulated, focusing on brake caliper suspension stiffness and wheel–rail contact geometry.

### 4.1. Vehicle System Dynamic Model

The actual metro vehicle consists of numerous complex components assembled through various nonlinear connections. When establishing a dynamic model, simplifications of the vehicle system are necessary. The following assumptions are made:

- The interactions between vehicles are not considered, and a single-vehicle model is established.
- Elastic deformation is not considered, and all components are regarded as rigid bodies.
- Traction and braking conditions are ignored, and the vehicle is assumed to operate at a constant speed on the track.
- The influence of curved tracks is ignored, and the track is set as a straight line.

**Figure 11:**
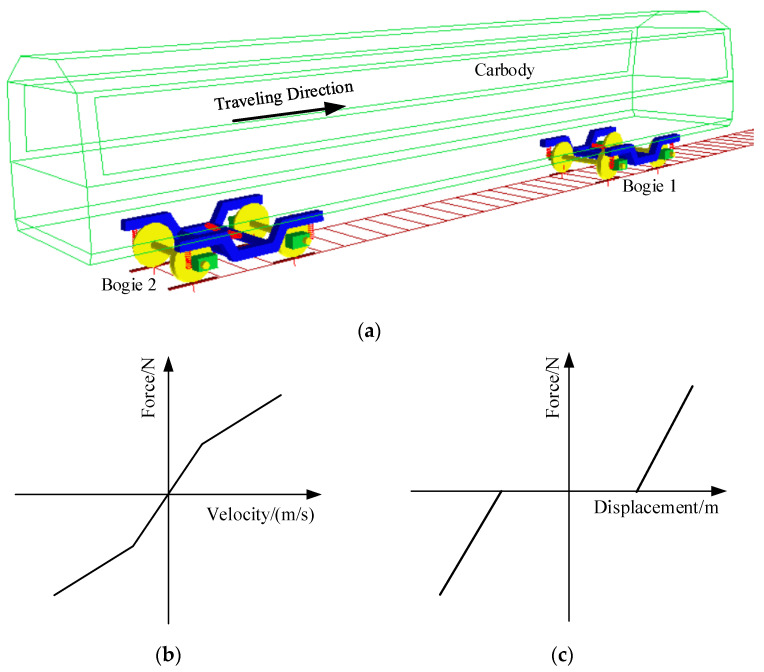
Vehicle system model; (**a**) scheme; nonlinear characteristics of (**b**) damper and (**c**) secondary lateral stop.

Based on the above assumptions, a dynamic model of the metro vehicle is established using SIMPACK software (software version: 8904). The vehicle model consists of one carbody, two bogies, four wheelsets, and eight axle boxes. In the Body module, rigid body models of the carbody, bogies, and wheelsets are created, and then they are set to move forward uniformly along the track centerline using Joint 7. The connections between axle boxes and bogies, and between bogies and the carbody, are made using Force elements, representing the primary and secondary suspensions. The primary suspension includes a swing-arm node, steel spring, and primary vertical damper, while the secondary suspension includes air springs, secondary lateral dampers, secondary lateral stop, anti-roll bars, and traction rods. The carbody, bogies, and wheelsets have six degrees of freedom (motion along the x, y, and z axes and rotation around the x, y, and z axes), while the axle boxes have only one degree of freedom (rotation around the y-axis). The metro vehicle dynamic model is illustrated in Figure 11a. Some main paraments of metro vehicles are shown in Table 3.

The actual metro vehicle system involves numerous nonlinearities, the most significant of which are the wheel–rail nonlinear contact and the nonlinearities in the suspension system. In the dynamic model, the wheel tread profile measured during the field test in Section 2.3 is used, and the rail profile is selected for the CN60. These wheel and rail profiles are input into SIMPACK, and the wheel–rail module generates the nonlinear contact parameters. The normal force between wheel and rail is calculated using Hertz contact theory, while the wheel–rail creep forces are calculated based on Kalker’s simplified theory.

The nonlinear characteristics in the vehicle suspension system primarily include a hydraulic damper and a secondary lateral stop. As shown in Figure 11b, the hydraulic damper exhibits nonlinear behavior: the slope is steep when the vibration velocity is below the damper’s relief speed, and it becomes gentler when the vibration velocity exceeds the relief speed. Figure 11c illustrates the nonlinear characteristics of the secondary lateral stop, which is described by a piecewise function with a dead zone. The stiffness is zero when the secondary lateral displacement is within the stop clearance. Once the displacement exceeds the stop clearance, the stiffness increases abruptly.

The track irregularities in the model are generated based on the American fifth-grade spectrum. The United States Department of Transportation Federal Railroad Administration (FRA) derived the track irregularity power spectral density from extensive empirical data, fitting it into an even-order function represented by cutoff frequency and roughness constant. Track irregularities are generated using this power spectral density function. According to the maximum operating speed, the American track spectrum is divided into six grades, with the fifth-grade spectrum having a maximum operating speed of 144 km/h.

**Figure 12:**
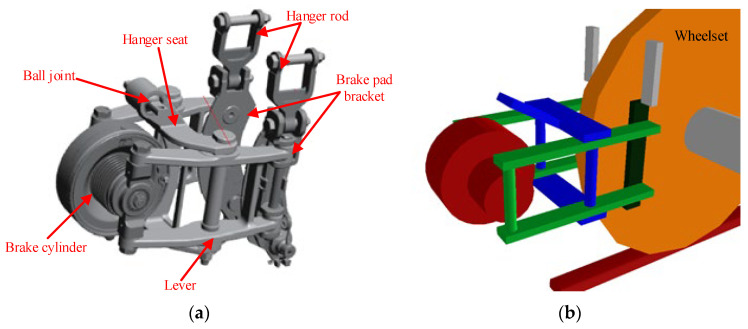
Model of brake caliper; (**a**) there dimensional model (**b**) dynamic model.

To investigate the geometric interference issue between the brake caliper and the wheelset caused by bogie hunting instability of metro vehicles, a dynamic model of the brake caliper was established. Illustrated in Figure 12a, the three-dimensional model of the brake caliper comprises essential components such as a hanger seat, brake cylinder, lever, brake pad bracket, and hanger rod. The rear section of the brake caliper is suspended from the frame via the hanger seat, connected through a ball joint mechanism. The lever, connected to the hanger seat by pivot pins on both sides, is linked to the brake cylinder at its rear end. At the front end of the lever, the brake pad bracket hosts the brake pads, while the brake pad bracket hanger suspends it freely from the frame, allowing for unrestricted movement. In non-braking situations, the deflated state of the brake caliper causes the brake cylinder to retract, opening the brake pad bracket through the lever. Conversely, during braking, inflation of the brake caliper expands the brake cylinder on both sides, facilitated by the lever, which closes the brake pad bracket. This engagement enables the brake pads to create braking force via friction against the brake disc on the wheelset. Figure 12b illustrates the dynamic model of the brake caliper, incorporating elements such as the brake cylinder, lever, suspension bracket, and brake pad bracket. The suspension bracket offers six degrees of freedom, while the lever, brake cylinder, and brake pad bracket each possess one degree of rotational freedom around the z-axis. The simulation of the interaction between the suspension bracket and the frame utilizes a 43-force element, considering parameters like axial stiffness, radial stiffness, rotational stiffness, and cardanic stiffness, as per the ball joint design.

### 4.2. Model Verification

After establishing the dynamic model of the metro vehicle, verifying the model’s accuracy is crucial for subsequent simulation analyses. This section will validate the model’s accuracy by comparing field test results with simulation results. If the test data and simulation results are consistent, the model is accurate; otherwise, it is unreliable. First, acceleration and displacement sensors are placed in the model built in Section 4.1, with the sensor positions matching those described in Section 2.2. Next, the wheel tread data measured in Section 3.1 is imported into the metro vehicle dynamic model. Track irregularities are generated based on the American fifth-grade spectrum. The dynamic model then runs at a speed of 100 km/h, the same as the maximum speed in the field test. Finally, the acceleration and displacement data obtained from the simulation are processed using the data processing methods described in Section 3.2.

**Figure 13:**
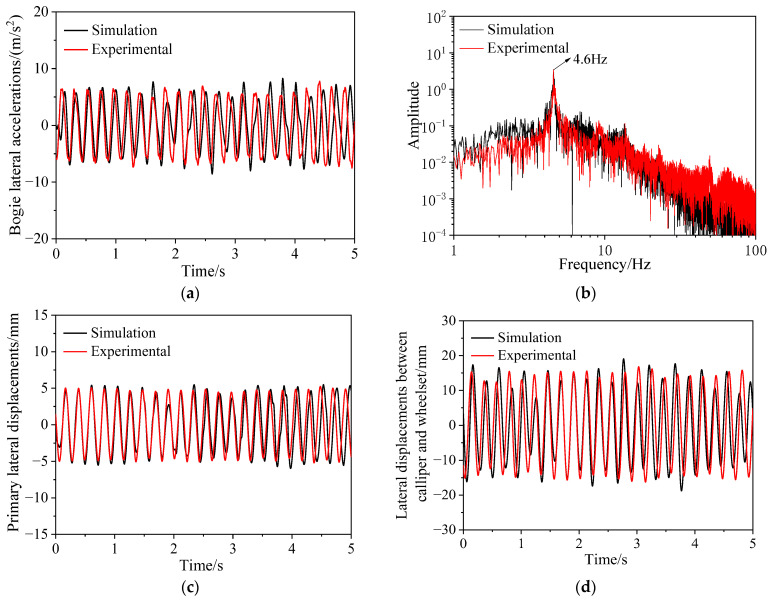
Comparison between field test and simulation; time history (**a**) and spectrogram (**b**) of lateral accelerations of bogie; (**c**) primary lateral displacements; (**d**) lateral displacements between caliper and wheelset.

The comparison between simulation results and test results is shown in Figure 13. First, the lateral acceleration signal above the left side of wheelset 1 is compared. Figure 13a and 13b show the time domain and frequency-domain of the lateral acceleration of the frame, respectively. It can be observed that the simulated and experimental lateral acceleration amplitudes are basically consistent, and the dominant vibration frequency is 4.6 Hz for both. Figure 13c shows the primary lateral displacement of the left of wheelset 1, where the amplitudes of the simulation and experimental results are consistent, and the waveforms overlap well. Figure 13d displays the relative lateral displacement between the brake caliper and the wheelset on the left side of wheelset 1, where the amplitudes of the simulation and experimental results are close, and the waveforms are generally aligned.

The comparison above shows that the simulation results of the dynamic model of metro vehicles established in Section 4.1 are consistent with the field test results, indicating that the model is accurate and reliable. In Section 4.3, this model will be utilized for simulated analysis to address the issues of bogie hunting instability and geometric interference between the brake caliper and the wheelset.

### 4.3. Simulation Analysis

The analysis of the field test results in Section 3 reveals the underlying cause of the geometric interference between the brake caliper and the wheel in metro vehicles, which stems from frame hunting. Despite the lateral displacement remaining modest at 5 mm during frame hunting, the lateral relative displacement between the brake caliper and the wheel surges to 15 mm, significantly exceeding the maximum displacement threshold. This stark difference underscores substantial movements of the brake caliper in relation to the frame during frame hunting episodes. Video documentation from the field tests vividly captures the substantial swinging motion of the brake caliper relative to the frame during these occurrences. It is this distinct phenomenon that drives the lateral relative displacement between the brake caliper and the wheel beyond the prescribed threshold, leading to the failure of geometric interference between the brake caliper and the wheel. To address this issue, this section delves into a comprehensive examination of the impact of brake caliper suspension stiffness on the swinging motion of the brake caliper. As explained in Section 3.1, the suspension bracket of the brake caliper is connected to the frame via a ball joint, which encompasses axial, radial, rotational, and cardanic stiffness, with the latter exerting the most significant influence on the brake caliper’s swinging angle.

**Figure 14:**
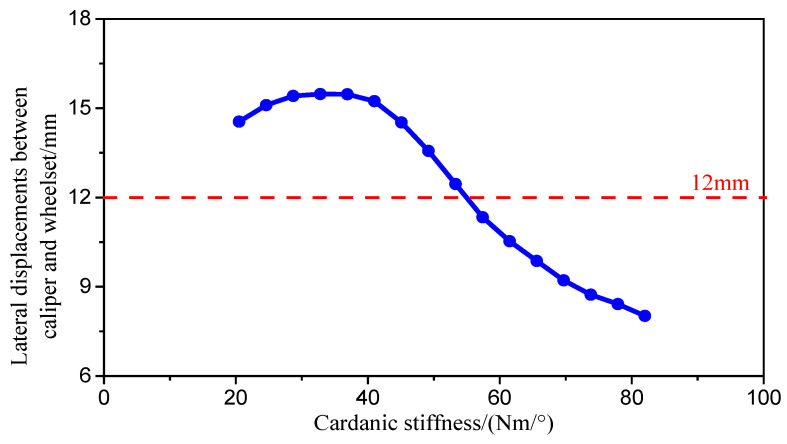
Effect of cardanic stiffness of caliper on lateral displacements between caliper and wheelset.

The vehicle system dynamic model is utilized to simulate the swing angle of the brake caliper under varying cardanic stiffness values by adjusting the ball joint’s cardanic stiffness. The lateral relative displacement between the brake caliper and the wheel is determined by multiplying the swing angle by the distance from the bottom of the brake caliper (which experiences the maximum lateral displacement during swinging, serving as the actual interference point) to the suspension point, and then adding the lateral displacement of the primary suspension. Figure 14 presents the simulation outcomes, indicating that when the cardanic stiffness is below 35 Nm/°, the lateral relative displacement between the brake caliper and the wheel increases as the cardanic stiffness rises. However, excessively low cardanic stiffness can adversely affect the brake caliper’s braking performance. Conversely, when the brake caliper’s cardanic stiffness surpasses 35 Nm/°, the lateral relative displacement decreases with increased swinging stiffness. To prevent geometric interference failure between the brake caliper and the wheel during operation, the lateral relative displacement must remain under 12 mm (considering the measured lateral gap between the brake caliper and the wheel on test vehicles ranging from 12 to 23 mm). Therefore, the cardanic stiffness of the brake caliper suspension ball joint should exceed 55 Nm/°.

Increasing the cardanic stiffness of the brake caliper aids in reducing swing amplitude, thus lowering the lateral relative displacement between the brake caliper and the wheel, potentially resolving their geometric interference issue. However, this adjustment does not address the bogie hunting problem resulting from high equivalent conicity.

**Figure 15:**
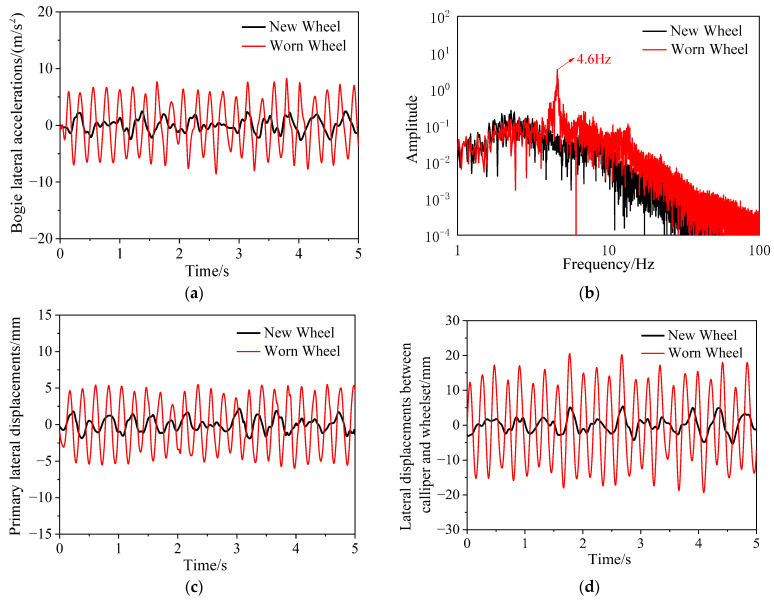
Comparison between new wheel and worn wheel; time history (**a**) and spectrogram (**b**) of lateral accelerations of bogie; (**c**) primary lateral displacements; (**d**) lateral displacements between caliper and wheelset.

The bogie hunting instability of metro vehicles is caused by the high equivalent conicity of the wheel–rail contact, which results from concave wear on the wheel tread. Wheel reprofiling is a method used to restore the wheel to its new tread condition. The vibration characteristics of metro vehicles are analyzed using a dynamic model under conditions of new and worn wheels. The track excitation is based on fifth-grade track irregularity PSD of American railways, with a vehicle operating speed of 100 km/h. Figure 15a,b compare the time and frequency domains of lateral acceleration of the frame between new and worn wheel conditions. It can be observed that the lateral acceleration of the frame under the new wheel condition has a small amplitude and exhibits random vibrations compared to the worn wheel condition. The frequency spectrum shows that the bogie hunting frequency does not appear under the new wheel condition. Figure 15c compares the primary lateral displacement, indicating a significant reduction in amplitude after wheel reprofiling. Figure 15d compares the lateral relative displacement between the brake caliper and the wheelset, showing a significant reduction in amplitude under the new wheel condition. This indicates that the brake caliper no longer experiences substantial lateral oscillations, and there is no geometric interference between the brake caliper and the wheelset.

In summary, wheel reprofiling effectively resolves the bogie hunting instability of metro vehicles and eliminates the geometric interference problem between the brake caliper and the wheelset caused by bogie hunting instability.

## 5. Conclusions

(1) The main cause of bogie hunting instability in metro vehicles is the high conicity of the wheel–rail contact resulting from wheel tread wear. Bogie hunting instability not only affects operational safety and ride comfort but also leads to geometric interference issues between the brake caliper and wheel.
(2) By processing the data collected by sensors in 2 s intervals, the real-time monitoring of the vehicle’s operational status is achieved. When the wheelset lateral force and comfort index exceed the limit, the vehicle speed should be reduced until the bogie returns to a stable state.
(3) Considering that metro vehicle bogie hunting instability mainly occurs under high conicity wheel–rail contact conditions after significant wheel tread wear, monitoring bogie hunting stability can be cost-effectively focused on metro vehicles in the later stages of wheel wear (after 300,000 km).
(4) Wheel reprofiling can effectively resolve bogie hunting instability. Considering wheel lifespan, the reprofiling cycle for metro vehicles should be set at 350,000 km.

It should be noted that the reprofiling of railway vehicle wheels should not only consider changes in the equivalent conicity. When issues such as scuffing, flat spots, or out-of-roundness occur, timely reprofiling is also necessary to address these problems. Meanwhile, suspension parameters also affect the hunting stability of railway vehicle bogies. Increasing the primary suspension stiffness can improve bogie hunting stability, but high stiffness can be detrimental to the vehicle’s ability to curve. Research into adaptive suspension systems is a future direction for study.

## Figures and Tables

**Table 1:** Specifications of the selected sensor.

Type	Installation Position	Model	Power Supply	Range	Voltage Sensitivity
Acceleration	Carbody floor	LC0709-5	5 VDC	±5 g ^1^	310 mV/g
	Bogie frame	LC0709-18	5 VDC	±18 g	100 mV/g
	Axle box	LC0707-100	5 VDC	±100 g	20 mV/g
Displacement	Between frame and wheelset	ILD2300-100	11–30 VDC	30–130 mm	40 mV/mm
	Between caliper and wheelset	ILD2300-250	11–30 VDC	50–300 mm	100 mV/mm

^1^ 1 g = 9.81 m/s^2^.

**Table 2:** Frequency correction factor.

Vertical		Lateral	
0.5–5.9 Hz	*F*(*f*) = 0.325*f*^2^	0.5–5.4 Hz	*F*(*f*) = 0.8*f*^2^
5.9–20 Hz	*F*(*f*) = 400/*f*^2^	5.4–26 Hz	*F*(*f*) = 650/*f*^2^
>20 Hz	*F*(*f*) = 1	>26 Hz	*F*(*f*) = 1

**Table 3:** Some main parameters of the metro vehicle.

Parameter	Value	Unit
Mass of carbody	20	t
Rotational inertia of carbody roll	108.12	t·m^2^
Rotational inertia of carbody pitch	772.52	t·m^2^
Rotational inertia of carbody yaw	701.49	t·m^2^
Mass of bogie frame	2972.77	kg
Rotational inertia of frame roll	1206	kg·m^2^
Rotational inertia of frame pitch	1736	kg·m^2^
Rotational inertia of frame yaw	2809	kg·m^2^
Mass of wheelset	1494	kg
Rotational inertia of wheelset roll	706	kg·m^2^
Rotational inertia of wheelset pitch	109	kg·m^2^
Rotational inertia of wheelset yaw	716	kg·m^2^
Bogie wheelbase	2300	mm
Distance between bogie center	12,600	mm
Primary vertical stiffness	1203	kN/m
Primary longitudinal stiffness	9000	kN/m
Primary lateral stiffness	3000	kN/m
Secondary vertical stiffness	0.245	kN/m
Secondary longitudinal stiffness	0.118	kN/m
Secondary lateral stiffness	0.118	kN/m
Radial stiffness of caliper	29,000	kN/m
Axial stiffness of caliper	4000	kN/m
Torsional stiffness of caliper	17	Nm/°
Cardanic stiffness of caliper	41	Nm/°

## Data Availability

Data are contained within the article.

## References

[1] Iwnicki S., Spiryagin M., Cole C., McSweeney T. (2019). Handbook of Railway Vehicle Dynamics.

[2] Kaiser I., Poll G., Voss G., Vinolas J. (2019). The impact of structural flexibilities of wheelsets and rails on the hunting behaviour of a railway vehicle. Veh. Syst. Dyn..

[3] Gao H., Sun J., Shiju E., Chi M. (2024). Cavitation induced hydraulic yaw damper failure and its effect on railway vehicle dynamic stability. Eng. Fail. Anal..

[4] Sharma R., Dhingra M., Pandey R.K., Rathore Y. (2015). Dynamic analysis of railway vehicles. J. Sci..

[5] Li Y., Chi M., Guo Z., Liang S. (2023). An abnormal carbody swaying of intercity EMU train caused by low wheel–rail equivalent conicity and damping force unloading of yaw damper. Rail. Eng. Sci..

[6] Li Y., Dai L., Guo Z., Chi M. (2024). Carbody abnormal lateral vibration failure of high–speed train induced by the coupling factor of the wheel re–profiling method and excessive rail wear. Eng. Fail. Anal..

[7] Fujimoto H., Miyamoto M. (1996). Measures to reduce the lateral vibration of the tail car in a high speed train. Proc. IMechE Part F J. Rail Rapid Transit.

[8] Sun J., Chi M., Jin X., Liang S., Wang J., Li W. (2021). Experimental and numerical study on carbody hunting of electric locomotive induced by low wheel–rail contact conicity. Veh. Syst. Dyn..

[9] Zheng B., Wei L., Zeng J., Huang C. (2024). Carbody hunting behavior of high speed vehicles in low effective conicity of wheel-rail contact. IMechE Part F J. Rail Rapid Transit..

[10] Liu C., Song Y., Li F., Wu P., Ye Y. (2023). Stress spectrum compilation method and residual life prediction for hot spot position of metro bogie frame under resonance condition. Eng. Fail. Anal..

[11] Guo Z., Chi M., Sun J., Li Y., Dai L. (2023). Experimental and numerical research on the bogie hunting of a high-speed train caused by the empty stroke of yaw damper. Veh. Syst. Dyn..

[12] Uyulan C., Gokasan M., Bogosyan S. (2019). Hunting stability and derailment analysis of the high-speed railway vehicle moving on curved tracks. Int. J. Heavy Veh. Syst..

[13] Bustos A., Tomas-Rodriguez M., Rubio H., Castejon C. (2023). On the nonlinear hunting stability of a high-speed train bogie. Nonlinear Dyn..

[14] Skerman D., Cole C., Spiryagin M. (2022). Determining the critical speed for hunting of three-piece freight bogies: Practice versus simulation approaches. Veh. Syst. Dyn..

[15] Uyulan C., Gokasan M., Bogosyan S. (2017). Dynamic investigation of the hunting motion of a railway bogie in a curved track via bifurcation analysis. Math. Probl. Eng..

[16] Wei L., Zeng J., Huang C., Zheng B., Li X. (2024). Hunting stability and dynamic stress analysis of a high-speed bogie using elastic-suspended motors as dynamic vibration absorber. Veh. Syst. Dyn..

[17] Zhang X., Wu G., Li G., Yao Y. (2020). Actuator optimal placement studies of high-speed power bogie for active hunting stability. Veh. Syst. Dyn..

[18] Yao Y., Wu G., Sardahi Y., Sun J. (2018). Hunting stability analysis of high-speed train bogie under the frame lateral vibration active control. Veh. Syst. Dyn..

[19] Shi H., Zeng J., Guo J. (2024). Disturbance observer-based sliding mode control of active vertical suspension for high-speed rail vehicles. Veh. Syst. Dyn..

[20] Mihailescu I., Popa G., Tudor E., Vasile I., Gheti M.A. (2023). Experimental Study of Wheel-to-Rail Interaction Using Acceleration Sensors for Continuous Rail Transport Comfort Evaluation. Sensors.

[21] Shan W., Wu P., Wu X., Zhang F., Shi H. (2019). Effect of wheel polygonization on the axle box vibration and bolt self-loosening of high-speed. J. Phys. Conf. Ser..

[22] Dumitriu M. (2022). Condition Monitoring of the Dampers in the Railway Vehicle Suspension Based on the Vibrations Response Analysis of the Bogie. Sensors.

[23] Palmer F.S., Luber B., Fuchs J., Kern T., Rosenberger M. Data-driven fault diagnosis of bogie suspension components with on-board acoustic sensors. Proceedings of the 5th European Conference of the Prognostics and Health Management Society.

[24] Lebela D., Soize C., Funfschilling C., Perrin G. (2020). High-speed train suspension health monitoring using computational dynamics and acceleration measurements. Veh. Syst. Dyn..

[25] Shi H., Wang J., Wu P., Song C., Teng W. (2018). Field measurements of the evolution of wheel wear and vehicle dynamics for high-speed trains. Veh. Syst. Dyn..

[26] (2019). Specification for Dynamic Performance Assessment and Testing Verification of Rolling Stock.

[27] (2008). Concerning a Technical Specification for Interoperability Relating to the ‘Rolling Stock’ Sub-System of the Trans-European High-Speed Rail System.

[28] Li Y., Dai H., Qi Y., Qu S., Sun Y. (2023). Experimental study of bogie instability monitoring and suppression measures for high-speed EMUs. Proc. IMechE Part F J. Rail and Rapid Transit..

[29] Kulkarni R., Qazizadeh A., Berg M., Carlsson U., Stichel S. (2022). Vehicle running instability detection algorithm (VRIDA): A signal based onboard diagnostic method for detecting hunting instability of rail vehicles. Proc. IMechE Part F J. Rail and Rapid Transit..

[30] Sun J., Meli E., Song X., Chi M., Jiao W., Jiang Y. (2023). A novel measuring system for high-speed railway vehicles hunting monitoring able to predict wheelset motion and wheel/rail contact characteristics. Veh. Syst. Dyn..

[31] Ning J., Cui W., Chong C., Ouyang H., Chen C., Zhang B. (2019). Feature recognition of small amplitude hunting signals based on the MPE-LTSA in high-speed trains. Measurement.

[32] (2004). Method for Determining the Equivalent Conicity, 1st ed..

[33] (2009). Testing and Approval of Railway Vehicles from the Point of View of Their Dynamic Behaviour–Safety–Track Fatigue–Running Behaviour, 4th ed..

[34] Wei L., Zeng J. (2014). Indirect method for wheel–rail force measurement and derailment evaluation. Veh. Syst. Dyn..

